# An integrated functional genomic study of acute phenobarbital exposure in the rat

**DOI:** 10.1186/1471-2164-11-9

**Published:** 2010-01-06

**Authors:** Claire L Waterman, Richard A Currie, Lisa A Cottrell, Jacky Dow, Jayne Wright, Catherine J Waterfield, Julian L Griffin

**Affiliations:** 1 Department of Biochemistry, University of Cambridge, Cambridge, CB2 1QW, UK; 2 Syngenta Central Toxicology Laboratory, Alderley Park, Macclesfield, Cheshire, SK10 4TJ, UK; 3 Current address: Syngenta, Jealott's Hill International Research Centre, Bracknell, Berkshire, RG42 6EY, UK

## Abstract

**Background:**

Non-genotoxic carcinogens are notoriously difficult to identify as they do not damage DNA directly and have diverse modes of action, necessitating long term in vivo studies. The early effects of the classic rodent non-genotoxic hepatocarcinogen phenobarbital have been investigated in the Fisher rat using a combination of metabolomics and transcriptomics, to investige early stage mechanistic changes that are predictive of longer term pathology.

**Results:**

Liver and blood plasma were profiled across 14 days, and multivariate statistics used to identify perturbed pathways. Both metabolomics and transcriptomics detected changes in the liver which were dose dependent, even after one day of exposure. Integration of the two datasets associated perturbations with specific pathways. Hepatic glycogen was decreased due to a decrease in synthesis, and plasma triglycerides were decreased due to an increase in fatty acid uptake by the liver. Hepatic succinate was increased and this was associated with increased heme biosynthesis. Glutathione synthesis was also increased, presumably in response to oxidative stress. Liquid Chromatography Mass Spectrometry demonstrated a remodeling of lipid species, possibly resulting from proliferation of the smooth endoplasmic reticulum.

**Conclusions:**

The data fusion of metabolomic and transcriptomic changes proved to be a highly sensitive approach for monitoring early stage changes in altered hepatic metabolism, oxidative stress and cytochrome P450 induction simultaneously. This approach is particularly useful in interpreting changes in metabolites such as succinate which are hubs of metabolism.

## Background

Non-genotoxic carcinogens are difficult to screen for as the development of tumours only occurs after long term exposure and, since there is no damage to DNA, they cannot be detected by conventional genotoxicity assays such as the Ames test, *in vitro *clastogenesis assays or the mouse micronucleus test. Definitive characterization of non-genotoxic rat carcinogens requires the outcome of a two year bioassay. There are earlier indicators of hepatocarcinogenic potential which include increased liver weight, evidence of cytotoxicity, enzyme induction and CAR agonist activity which are used to aid dose setting for long term bioassays and in estimating risk. However, their predictive power is limited and complicated by the fact that non-genotoxic carcinogens represent a diverse range of mechanisms [1,2].

Phenobarbital (PB) is a well characterized rodent non-genotoxic carcinogen which causes an increase in the incidence of liver tumors after long term exposure. This follows a reversible increase in liver weight, attributed to a combination of hyperplasia and hypertrophy [3,4]. In Fischer F344 rats exposed to PB in the diet for two years, an increase in the incidence of neoplastic nodules was reported at a dose of 1000 ppm [5], with no changes observed at 500 or 600 ppm [6,7]. In another study Rossi *et al*. found an increase in adenomas in Wistar rats exposed to 500 ppm PB in drinking water following exposure for three years [8]. Isenberg *et al*. reported that in F344 rats, exposure to 500 ppm PB in the diet for 1-2 weeks produces an increase in relative liver weight which is reversible on returning the animals to control diet. If the exposure is continued for 18 months, then a sustained increase in relative liver weight is observed. However, the increase in liver weight is only accompanied by a transient increase in DNA synthesis, peaking at two weeks of exposure and no hepatic cancer [9].

With the advent of global profiling approaches, including transcriptomics, proteomics and metabolomics, there is now the opportunity to profile different levels of organization of a biological system in tissues and biofluids, thus aiding the understanding of mechanisms/modes of action and the identification of potential biomarkers of responses which reflect underlying pathophysiological perturbations. In addition these 'omic' approaches are hypotheses generating and are particularly appropriate for systems where the mechanism of toxicity is unknown or poorly defined [10-12]. Furthermore, profiling a range of potential biomarkers may provide more sensitive or earlier indicators of pathological change.

In the present study a combination of metabolomics, transcriptomics and histology were used to identify changes occurring in the liver and plasma of Fisher F344 rats during exposure to a range of doses of PB over a period of 14 days. The doses were selected to provide a low dose of 50 ppm which is non-carcinogenic and does not cause an increase in liver weight, a middle dose of 500 ppm which causes an increase in liver weight but is not carcinogenic and high dose of 1000 ppm which is carcinogenic and causes an increase in liver weight. The integration of data from different 'omic' techniques presents a considerable challenge, but by combining datasets as part of a data fusion process, it is possible to produce information of greater value than that obtained from the individual datasets. This study demonstrates how this data fusion can be achieved in order to enhance understanding of the mechanisms involved in a toxic response.

## Results

### Liver and body weight

There was a significant increase in liver weight (adjusted for body weight) in PB treated animals compared to controls (table 1) at all time points for 500 and 1000 ppm. Body weight was significantly increased in the 500 and 1000 ppm groups at day 14 (p < 0.01), reflecting an increase in the average food intake for the 500 and 1000 ppm groups (10%and 11%, respectively; p < 0.01).

**Table 1:** Liver and body weights, Ki67 labeling indices and plasma clinical biochemistry

		Dose PB			
		
		Control	50 ppm	500 ppm	1000 ppm
**Day 1**	Relative liver weight (%)	4.57 ± 0.17	4.67 ± 0.12	4.89 ± 0.09**	4.58 ± 0.15
	Body weight (g)	205.8 ± 3.6	207.0 ± 6.9	210.2 ± 9.0	207.2 ± 8.8
	Ki67 labeling index (%)	6.77 ± 2.54	8.63 ± 2.56	7.94 ± 2.41	9.87 ± 2.88
	Total Protein (g/l)	62.9 ± 1.1	62.7 ± 1.7	63.9 ± 1.8	62.8 ± 2.0
	Cholesterol (mmol/l)	1.39 ± 0.06	1.35 ± 0.08	1.41 ± 0.11	1.39 ± 0.12
	Triglycerides (mmol/l)	1.27 ± 0.26	1.20 ± 0.07	1.56 ± 0.17	1.40 ± 0.44
	Alkaline Phosphatase (IU/l)	1120 ± 27	1071 ± 35	1117 ± 103	1048 ± 106
	Glutamate Dehydrogenase (U/l)	5.56 ± 0.48	6.62 ± 1.29	6.62 ± 1.55	5.64 ± 0.61

**Day 3**	Relative liver weight	4.73 ± 0.25	4.97 ± 0.21	5.17 ± 0.27*	5.63 ± 0.21**
	Body weight (g)	216.4 ± 10.4	214.8 ± 11.6	217.0 ± 7.8	209.0 ± 12.2
	Ki67 labeling index (%)	6.92 ± 0.73	6.14 ± 0.74	9.65 ± 0.58**	11.13 ± 1.73**
	Total Protein (g/l)	63.7 ± 3.7	62.9 ± 0.6	63.6 ± 3.6	64.8 ± 3.6
	Cholesterol (mmol/l)	1.38 ± 0.09	1.52 ± 0.15	1.72 ± 0.14*	1.86 ± 0.34**
	Triglycerides (mmol/l)	1.27 ± 0.17	1.29 ± 0.35	1.36 ± 0.25	1.33 ± 0.42
	Alkaline Phosphatase (IU/l)	1125 ± 112	1081 ± 38	942 ± 142	864 ± 133**
	Glutamate Dehydrogenase (U/l)	6.28 ± 0.53	5.43 ± 0.36	5.54 ± 0.94	5.82 ± 0.79

**Day 7**	Relative liver weight	4.31 ± 0.13	4.66 ± 0.12**	5.37 ± 0.17**	5.7 ± 0.14**
	Body weight (g)	225.6 ± 4.7	229.8 ± 12.1	228.8 ± 8.3	231.4 ± 6.7
	Ki67 labeling index (%)	5.08 ± 0.33	4.33 ± 1.12	4.63 ± 1.12	4.35 ± 1.21
	Total Protein (g/l)	61.0 ± 0.8	61.5 ± 1.1	64.3 ± 1.7**	66.3 ± 1.7**
	Cholesterol (mmol/l)	1.44 ± 0.10	1.41 ± 0.12	1.74 ± 0.07**	1.92 ± 0.08**
	Triglycerides (mmol/l)	1.04 ± 0.28	1.26 ± 0.11	0.90 ± 0.26	0.85 ± 0.07
	Alkaline Phosphatase (IU/l)	1007 ± 60	986 ± 95	883 ± 48*	837 ± 51**
	Glutamate Dehydrogenase (U/l)	4.70 ± 0.12	5.78 ± 0.43*	6.26 ± 0.79**	6.64 ± 0.52**

**Day 14**	Relative liver weight	4.22 ± 0.16	4.43 ± 0.18	5.39 ± 0.23**	5.7 ± 0.16**
	Body weight (g)	232.2 ± 7.8	240.6 ± 6.5	258.0 ± 11.4	266.2 ± 13.7
	Ki67 labeling index (%)	2.03 ± 1.04	1.10 ± 0.26	1.38 ± 0.49	1.83 ± 0.63
	Total Protein (g/l)	60.9 ± 0.9	59.8 ± 1.5	64.5 ± 1.9*	65.3 ± 3.2**
	Cholesterol (mmol/l)	1.22 ± 0.07	1.14 ± 0.10	1.42 ± 0.13*	1.60 ± 0.17**
	Triglycerides (mmol/l)	1.15 ± 0.11	1.23 ± 0.20	0.68 ± 0.10**	0.53 ± 0.14**
	Alkaline Phosphatase (IU/l)	952 ± 55	889 ± 149	821 ± 39	765 ± 14**
	Glutamate Dehydrogenase (U/l)	5.76 ± 1.26	6.56 ± 2.37	5.06 ± 0.86	6.92 ± 0.86

Relative liver weight, body weight, Ki67 labeling index and plasma clinical biochemistry data (for parameters which were significantly changed). All values are mean ± standard deviation. * p < 0.05 compared to time matched controls, ** p < 0.01 compared to time matched controls.

### Histopathology

**Figure 1:**
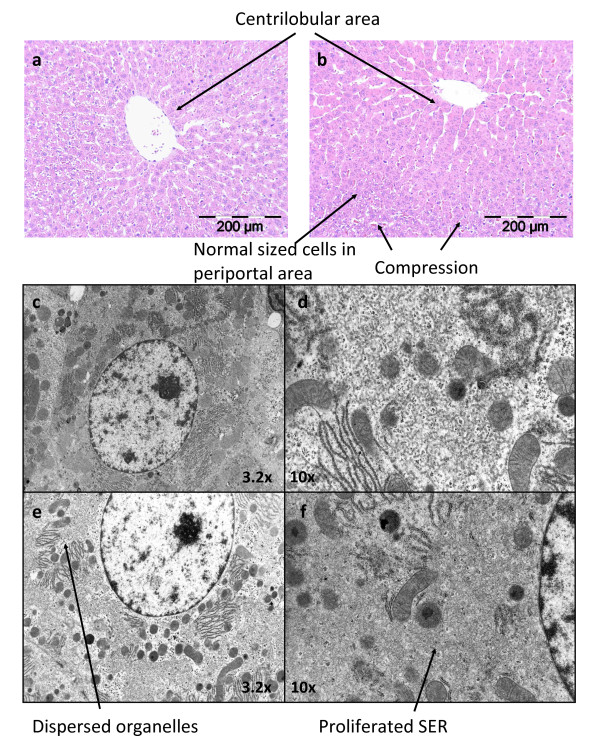
**Liver histopathology**. Liver histology. Upper panel: light microscopy image of hematoxylin and eosin stained liver tissue from control (**A**) and PB treated (**B**) animals. Livers from PB treated animals show centrilobular hypertrophy, compression of the periportal areas and eosinophilc appearance. Lower panel: electron microscopy images of control (**C**) and PB treated livers (**D**, **E & F**). PB treated livers showed dispersion of organelles and proliferation of the smooth endoplasmic reticulum.

Centrilobular hepatocyte hypertrophy was observed in all dose groups. The incidence and time of onset were related to dose. On day 1, only the mid dose (3/5) and high doses (1/5) were affected, whilst by day 3 the incidence had risen to 3/5 at 50 ppm, 5/5 at 500 ppm and 1000 ppm. Electron microscopy confirmed the hypertrophy to reflect smooth endoplasmic reticulum proliferation (figure 1).

### Clinical chemistry

Significant changes in plasma clinical chemistry were only detected with the 500 and 1000 ppm dose groups, with the exception of glutamate dehydrogenase where changes were also detected for the 50 ppm dose group. Plasma cholesterol and alkaline phosphatase activity were increased on days 3, 7 and 14. Triglycerides were decreased on day 14 and glutamate dehydrogenase was increased at day 7. Total protein was increased on days 7 and 14 (table 1).

### Labeling Index (Ki67)

At day 3 the Ki67 labeling indices were statistically significantly increased by doses of 500 (39%) and 1000 ppm (61%) (table 1). There was no change in the labeling index at other time points.

### Metabolomic analysis of liver tissue

**Figure 2:**
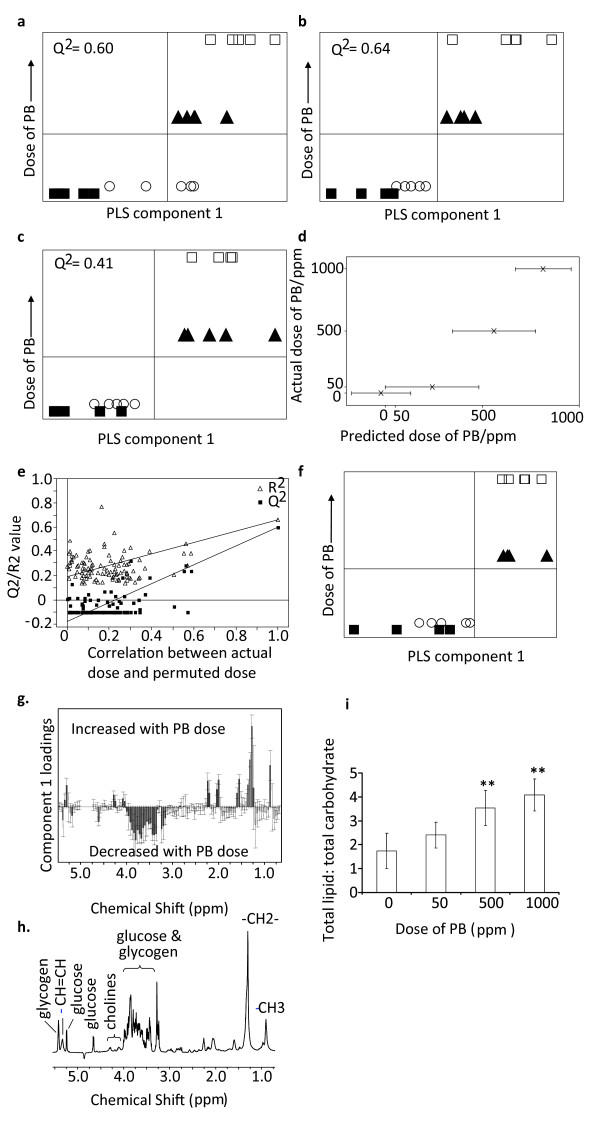
**PLS modeling of metabolomic data**. PLS plots correlating metabolic profiles with dose of PB at day 14 using ^1^H-NMR (A), GC-MS analysis of the aqueous extract (B), and GC-MS analysis of the lipid extract (C). In addition to the use of Q^2 ^values, models were validated to prevent over fitting by leaving samples out of the model building stage and then predicting their dose of PB (D) (mean ± standard deviation of 5 iterations) and also by using the validate function in SIMCA-P+ with 200 iterations (E). In this approach the actual model is compared with models formed where the Y value (in this case dose) is randomly permuted to produce models where there should be no correlation between the X-block and Y-block matrices. As demonstrated in panel E, the decreases in R^2 ^and Q^2 ^as the dose is randomly permuted is indicative of a robust model. PLS analysis of MAS ^1^H-NMR data separated the control and low dose from the mid and high dose groups (F). The corresponding loadings column plot (G i) and assigned spectra (G ii) showed that exposure to PB increased lipids and decreased sugars. The ratio of total lipid to total carbohydrate was increased in the mid and high dose groups compared to the control group (H). Plot show average ± standard deviation (n = 5) with significant changes as determined by ANOVA with Dunnett's post test for multiple comparisons labelled ** (p < 0.01). Key: Control (filled square) 50 ppm (open circle) 500 ppm (filled triangle) 1000 ppm (open circle).

Principal Components Analysis (PCA) of the ^1^H nuclear magnetic resonance (NMR) spectroscopy dataset from all time points combined demonstrated some separation according to dose (data not shown). However, analysis of individual time points using PCA demonstrated separation between the different dose groups which was improved by using partial least squares (PLS) to regress metabolic profile against dose of PB (Q^2 ^= 0.69, 0.54, 0.60, 1 component, for days 1, 3, and 14 respectively, Q^2 ^= 0.60, 2 components, day 7; figure 2A, table 2). The largest change associated with these PLS plots was a dose dependent decrease in glucose and glycogen at all time points in rats exposed to PB. There was also an increase in succinate and a decrease in adenosine with increasing dose of PB at all time points. At days 1-7, PB caused an increase in glutamine and glutathione (see additional file 1).

**Table 2:** Q^2 ^values for PLS models

	NMR aqueous	GC-MS aqueous	GC-MS lipid	Plasma NMR
**Day 1**	0.69 (1)	0.50 (1)	-	-

**Day 3**	0.54 (1)	0.55 (1)	0.43 (1)	0.35 (1)

**Day 7**	0.50 (2)	0.73 (1)	0.55 (1)	0.61 (2)

**Day 14**	0.60 (1)	0.64 (1)	0.41 (1)	0.68 (1)

Q^2 ^values for PLS models using data from different platforms and at different time points. The Q^2 ^is a measure of how robust a model is, scored out of 1, with higher values indicating a better model. The number in brackets is number of components in the model.

At each time point PCA of the data produced by GC-MS (gas chromatography-mass spectrometry) analysis of the aqueous phase separated the different doses of PB, although at days 1 and 3 the 50 ppm dose group could not be separated from the control group. The same trend was also detected using PLS to regress metabolic profile against dose with improved separation (Q^2 ^= 0.50, 0.55, 0.73, 0.64, all 1 component, days 1, 3, 7 and 14, respectively; figure 2B, table 2). PB caused an increase in succinate and glycine at all time points and 5-oxoproline at days 1, 3 and 14. Disaccharides, ethanolamine and fructose were also all decreased by PB at days 1, 3 and 7 and ribose was decreased at days 3 and 14 (table 3).

**Table 3:** Fold change in expression of selected genes.

	Fold change relative to time matched controls											
**Time point**	**Day 1**			**Day 3**			**Day 7**			**Day 14**		

**Dose (ppm)**	**50**	**500**	**1000**	**50**	**500**	**1000**	**50**	**500**	**1000**	**50**	**500**	**1000**

hexokinase D	1.10	0.67	0.23*	0.99	0.84	0.41	0.85	0.30*	0.39*	0.64	0.44*	**0.40**
glutamine synthetase	1.09	0.60**	0.57**	0.83	0.48**	0.39**	1.01	0.46*	0.45**	0.80*	0.44**	0.30*
glutamine cysteine ligase	1.00	1.28	1.59*	1.11	1.76**	1.80**	0.64**	0.69**	0.92	1.06	1.16	1.04
glutathione reductase	1.14	1.30*	1.57**	1.16	1.86**	1.99**	0.79	1.33**	1.74**	0.94	1.43	1.61*
malic enzyme	1.38	1.84**	1.72*	0.77	1.64*	1.87**	0.87	1.13	1.71	1.19	2.15*	1.74
aminolevulinate synthase	1.84	2.54*	3.68**	1.06	1.77*	2.29**	1.26	1.54	2.05	0.83	1.40	1.39
lipoprotein lipase	0.83*	0.95	1.19	1.13	1.69	2.32**	1.13	3.94**	7.33**	0.81	4.19**	5.16**
epoxide hydrolase	1.36*	2.19**	2.57**	1.14	2.46**	2.88**	1.15	2.38**	2.67**	1.72*	3.33**	3.33**
CYP 3A 3	1.21	1.66**	1.74**	0.97	1.71**	1.84**	1.02	1.69**	1.70**	1.04	1.58**	1.81**
Aldehyde dehydrogenase 1 A1	1.41	2.69*	4.87**	1.28	6.09*	9.82**	1.31	8.70**	14.11**	3.93	23.86**	24.59**
liver UDP-glucuronosyltransferase, PB-inducible form	1.88	3.96**	4.98**	2.77**	6.45**	6.71**	3.97**	6.18**	6.63**	3.63**	6.10**	5.89**
glutathione S-transferase Yb4 gene	0.91	1.73	2.19	1.65	6.27**	9.01**	1.45	6.19**	8.44**	1.80	6.56**	7.89**
CYP 2B2	4.23*	7.30**	7.06**	5.74**	8.75**	9.16**	5.58**	8.08**	7.01**	6.19**	8.48**	8.71**

Fold changes in expression for selected genes. * p < 0.05 compared to time matched controls, ** p < 0.01 compared to time matched controls.

While there was no dose dependent trend in the data from the lipid fraction detected by PCA or PLS at day 1, at days 3, 7 and 14 there was separation according to dose with the same trend being detected at each time point. By PCA there was some separation between the control and 50 ppm groups and the 500 and 1000 ppm groups which was increased by PLS but the control group and 50 ppm dose group were still poorly discriminated, as were the 500 and 1000 ppm dose groups (Q^2 ^= 0.43, 0.55, 0.41, all 1 component, days 3, 7 and 14 respectively; figure 2C, table 2). These models determined that PB caused an increase in oleic acid at days 3, 7 and 14 and an increase in cis-5,8,11,14,17-eicosapentaenoic acid at days 7 and 14. At days 3 and 7 PB caused a decrease in arachidonic acid, docosahexaenoic acid and pentadecanoic acid (table 3). The PLS models built for each technique and time point all passed cross-validation either in terms of predicting the dose of PB during a leave-one-out analysis (figure 2d) or during a random permutation test for dose (figure 2e).

### High resolution magic angle spinning ^1^H NMR analysis of liver tissue

Following the observation that PB exposure decreased the glucose and glycogen content of the liver, we hypothesized PB alters the ratio of mobile cytosolic lipid to carbohydrate in the liver. High resolution magic angle spinning (HRMAS) NMR spectroscopy is a technique which allows the acquisition of high resolution NMR spectra from solid tissue samples, and was conducted on intact liver samples from day 14 to determine if the ratio of lipid to carbohydrate in the liver was changed by PB exposure. PLS separated the 0 and 50 ppm groups from the 500 and 1000 ppm groups (Q^2 ^= 0.86, 4 components) (figure 2f). The loadings showed that resonances corresponding to the fatty acids and choline groups of lipids were increased with increasing dose of PB, whereas resonances from glucose and glycogen were decreased with increasing dose of PB (figure 2g). The ratio of total fat to total carbohydrate increased by 103% and 134% for the 500 and 1000 ppm groups, respectively (p < 0.01) (figure 2h).

### Metabolic profiling of plasma

**Figure 3:**
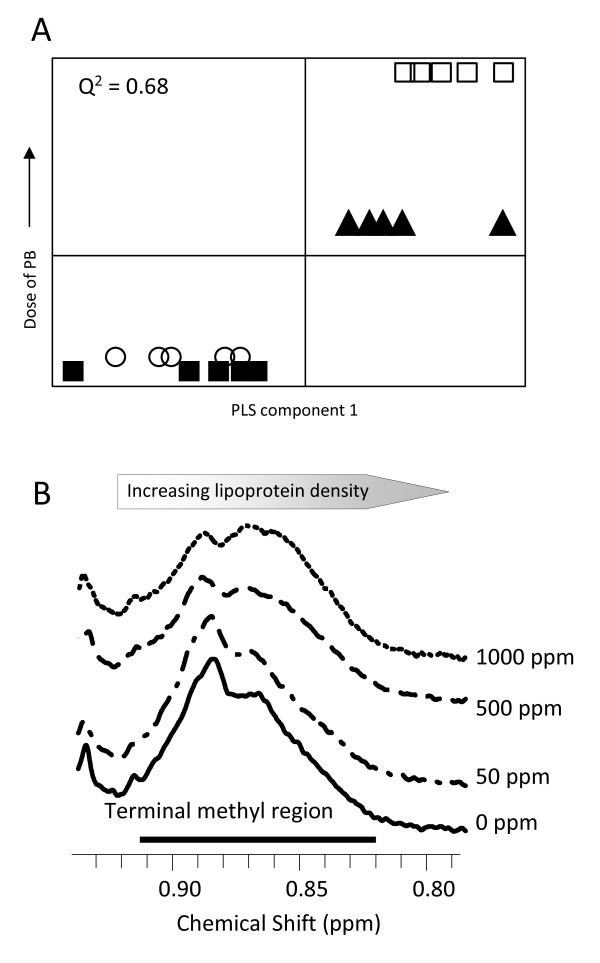
**Plasma lipoprotein profile**. PLS plots correlating metabolic profiles with dose of PB at day 14 using ^1^H-NMR spectroscopy of blood plasma (A). Key: Control (filled square), 50 ppm (open circle), 500 ppm (filled triangle), 1000 ppm (open square). Overlaid ^1^H-NMR spectra from the region containing the terminal methyl group of fatty acids in blood plasma (**B**). A change in the shape of the peak, with an increase in the proportion of lower chemical shift, higher density lipoprotein with increasing dose of PB is clearly detected. This is characteristic of an increase in high density lipoprotein particles relative to low density lipoprotein particles.

PCA of data from individual time points from the ^1^H-NMR spectroscopy of the blood plasma samples separated the control and 50 ppm dose groups from the 500 and 1000 ppm dose groups for all time points after day 1, with this separation becoming more pronounced at 7 and 14 days (PLS models Q^2 ^= 0.35, 0.68, 1 component, days 3 and 14 respectively, Q^2 ^= 0.61, 2 components, day 7; figure 3A, table 2). The concentrations of valine, lactate, glutamate and glutamine were increased by PB exposure at days 3, 7 and 14. Similarly, the concentrations of isoleucine, leucine acetate and choline/phosphocholine were increased at days 7 and 14. PB caused a decrease in plasma lipid which was more pronounced at later time points (table 3). The broad resonance corresponding to the terminal methyl groups of fatty acids changed in shape with an increase in the 0.84-0.86 ppm region and decrease in the 0.88-0.90 ppm region (figure 3B), reflecting an increase in the fraction of higher density lipoproteins [13].

### Gene expression profiling of liver

**Figure 4:**
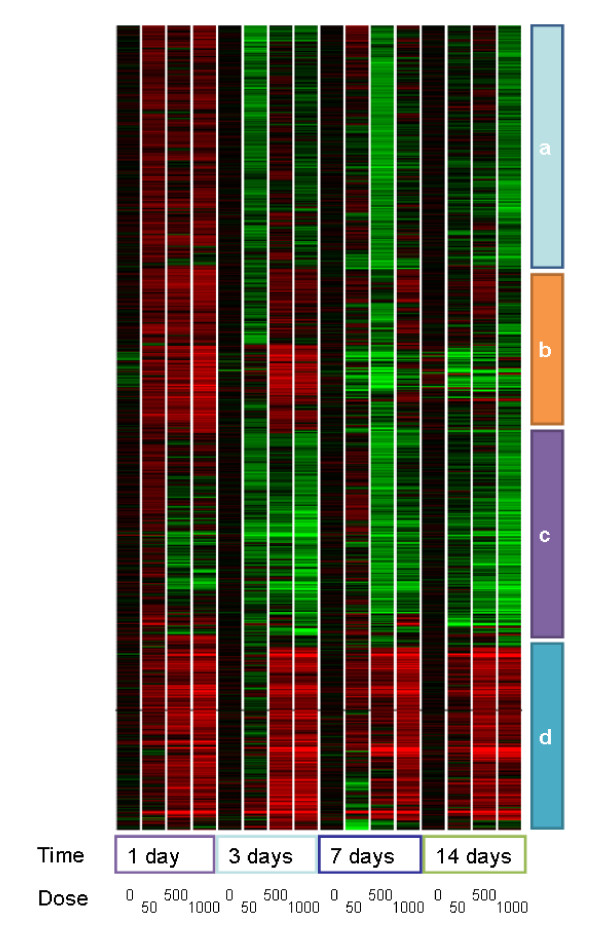
**Hierarchical clustering of transcriptomic data**. Hierarchical clustering using Pearson correlation coefficients revealed different temporal patterns of gene expression changes due to exposure to phenobarbital. Some genes were transiently upregulated at early time points (**A **&**B**), whereas others showed a sustained decrease (**C**) or increase (**D**) in expression.

Phenobarbital induced changes in the expression of 897 probesets. Hierarchical clustering using Pearson correlation coefficients revealed a complex temporal and dose-dependent pattern (figure 4). Some genes were clearly upregulated transiently at early time points (figure 4A and 4B), whereas others showed persistent down- or up-regulation (figure 4C and 4D respectively). Those genes showing transient increases in expression were over-represented in Gene Ontology, KEGG and GenMAPP annotations associated with the proteosome, starch and sucrose metabolism and the cell cycle. Those genes that have sustained alterations in expression were over-represented in genes with annotations including control of apoptosis, oxidative stress, and xenobiotic metabolism (additional file 2).

### Integration of metabolomic and transcriptomic data

**Figure 5:**
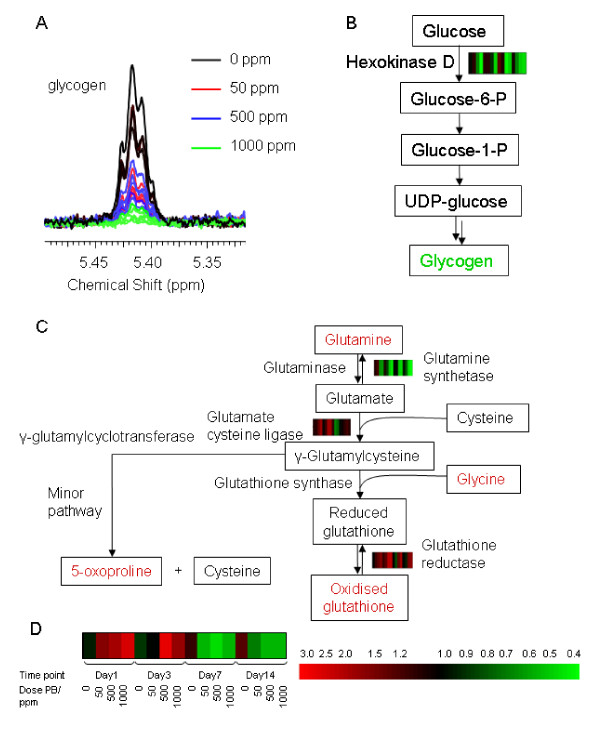
**Integration of metabolomic and transcriptomic data using pathway mapping**. Overlaid ^1^H-NMR spectra from day 14 samples showing a dose dependent decrease in glycogen. A similar response is seen at all other time points (**A**). The glycogen synthesis pathway demonstrating the correlation between transcriptional and metabolic changes. Hexokinase D, which is involved in control of the rate of glycogen synthesis, is decreased indicating that the decrease in glycogen is due to decreased synthesis (**B**). Glutathione synthesis pathway. Due to the extraction procedure, all glutathione detected is oxidized [38]. The increase in glutathione at days 1, 3 and 7 is likely to be due to an increase in glutamate cysteine ligase expression (**C**). The key to the gene expression data is given in (**D**).

Two approaches were taken to integrate the data generated by metabolomic and genomic analyses. Firstly, significant changes in gene expression were mapped on to the Kyoto Encyclopedia of Genes and Genomes (KEGG) metabolic pathways using GeneSpring (Agilent Technologies, Santa Clara, CA). This allowed the identification of pathways where both the expression of transcripts and concentration of metabolites were altered by PB. In this way an association between the decrease in hepatic glycogen and a decrease in glucokinase (hexokinase D) on the glycogen synthesis pathway was identified (figures 5A and 5B, table 3). This approach also identified a perturbation of glutathione metabolism in response to PB. Glutathione, glutamine, glycine and 5-oxo-proline were all increased by PB exposure. Glutamine cysteine ligase and glutathione reductase expression were increased whereas glutamine synthetase expression was decreased (figure 5C, table 3).

**Figure 6:**
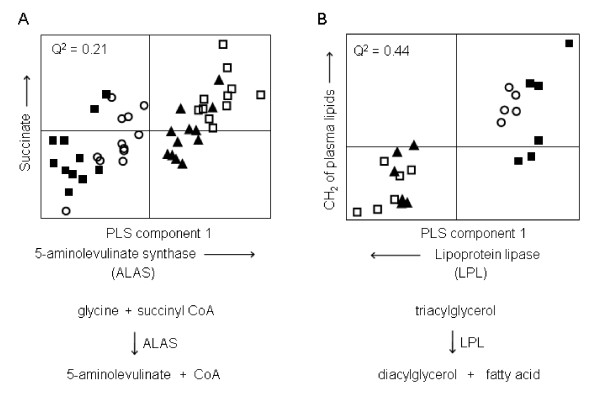
**Integration of metabolomic and transcriptomic data using PLS**. Partial Least Squares (PLS) plots modeling changes in gene expression associated with hepatic succinate at all time points (**A**) and CH_2 _of plasma lipids at days 7 and 14 (**B**), both measured by ^1^H-NMR spectroscopy. The increase in succinate detected with increasing dose of PB correlates with an increase in 5-aminolevulinate synthase (ALAS) expression and the decrease in plasma lipids detected with increasing dose of PB correlates with an increase in lipoprotein lipase (LPL) expression. Control (filled sqaure), 50 ppm (open circle), 500 ppm (filled triangle), 1000 ppm (open square).

In the second approach, PLS was used to build regression models using the genomic data as the X block and the normalized integrated areas of peaks corresponding to individual metabolites as the Y variable. In many cases an association was identified between PB induced metabolic changes, metabolizing enzymes, and enzymes involved with protecting against damage from oxidative stress. Epoxide hydrolase, glutathione S-transferase, glutathione reductase, cytochrome P450 2B2, cytochrome P450 3A1, aldehyde dehydrogenase and UDP-glucuronosyltransferase were all induced by PB (table 3). However, in the case of the increase in succinate detected at each time point by both GC-MS and ^1^H-NMR, the enzyme 5-aminolevulinate synthase (ALAS) was found to correlate with hepatic succinate (figure 6A, table 3). PLS also identified a correlation between decreased plasma lipids and increased hepatic lipoprotein lipase (LPL) at days 7 and 14 (figure 6B) and confirmed the association between decreased in glucokinase expression and decreased hepatic glycogen.

### PB induced remodeling of lipids

GC-MS can only provide information about the total fatty acid content of a sample, as information about the lipid species which originally contained the fatty acids is lost in the derivatisation procedure. Direct infusion ESI-MS was used to profile intact lipid species and this technique identified many changes in response to PB (Additional file 3). Using a Perl script the data was summarised in terms of a particular fatty acid or lipid class that had changed in the multivariate analysis of the data. In the presentation of the data it was not possible to determine definitively the position of the double bonds within given fatty acids by DI-ESI and so they are defined in terms of the number of carbons and degree of desaturation within the fatty acid, while presumed names are given in parentheses. However, where assignment was possible the name is given first.

**Figure 7:**
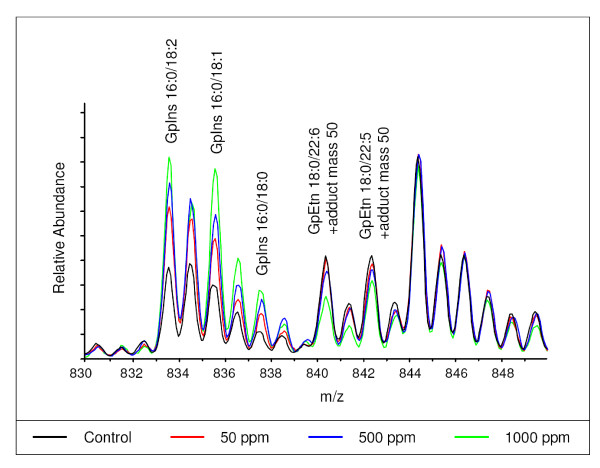
**Lipidomic analysis of the liver tissue**. Overlaying typical direct infusion ESI-MS spectra from the control, 50 ppm, 500 ppm and 1000 ppm groups at day 14 illustrates some of the changes occurring in the intact lipids. The 16:0 containing GpIns species are increased by exposure to PB and GpEtn species containing 18:0 in combination with a HUFA are decreased by exposure to PB. The GpEtn species are observed as adducts with an unknown species of mass 50.

Considering the free fatty acids, monoacylphospholipids and diacylphospholipids together there was a decrease in arachidonic acid (20:4) containing species at all time points. GpEtn and GpCho species were decreased at all time points and GpIns species were increased at all time points (Figure 7). There were also smaller decreases in species containing palmitic acid (16:0) at days 7 and 14 and 22:6 (docosahexaenoic acid) at days 3, 7 and 14 (Table 4). Many changes were observed in the free fatty acids detected by direct infusion ESI-MS. Stearic acid (18:0), oleic acid (18:1), 18:2, 20:3 and 20:5 (eicosapentaenoic acid) were all increased by PB at all time points and arachidic acid (20:0), 22:0 and 22:4 were decreased at all time points. Despite decreases in docosahexaenoic acid (22:6) and arachidonic acid (20:4) in the total lipid content as measured by GC-MS and direct infusion ESI-MS, no significant changes were seen for22:6 (docosahexaenoic acid) as the free fatty acid, and 20:4 (arachidonic acid) free fatty acid was only significantly decreased on day 7. However, decreases in diacylphospholipids containing these fatty acids were observed (Table 4).

**Table 4:** Number of occurrences of fatty acids and types of phospholipids in the lipid species significantly changed in response to PB.

	Day 3		Day 7		Day 14	
**Change with increasing dose of PB**	**Relative increase**	**Relative decrease**	**Relative increase**	**Relative decrease**	**Relative increase**	**Relative decrease**
14:0	0 (0/0/0)	0 (0/0/0)	0 (0/0/0)	0 (0/0/0)	0 (0/0/0)	1 (1/0/0)
16:0	11 (0/2/9)	7 (0/0/7)	3 (0/0/3)	18 (1/2/15)	10 (0/2/8)	18 (1/3/14)
16:1	2 (1/0/1)	0 (0/0/0)	0 (0/0/0)	1 (0/0/1)	0 (0/0/0)	3 (0/1/2)
17:0	0 (0/0/0)	2 (1/0/1)	0 (0/0/0)	2 (1/0/1)	0 (0/0/0)	0 (0/0/0)
18:0	19 (1/5/13)	12 (0/0/12)	18 (1/2/15)	13 (0/0/13)	20 (1/4/15)	15 (0/2/13)
18:1	6 (1/0/5)	5 (0/1/4)	4 (1/0/3)	5 (0/0/5)	4 (1/1/2)	7 (0/0/7)
18:2	7 (1/1/5)	2 (0/0/2)	6 (1/1/4)	6 (0/1/5)	5 (1/1/3)	5 (0/0/5)
18:3	1 (1/0/0)	0 (0/0/0)	0 (0/0/0)	1 (0/0/1)	0 (0/0/0)	1 (0/0/1)
19:0	0 (0/0/0)	1 (0/0/1)	0 (0/0/0)	1 (0/0/1)	0 (0/0/0)	0 (0/0/0)
20:0	0 (0/0/0)	1 (1/0/0)	0 (0/0/0)	1 (1/0/0)	0 (0/0/0)	1 (1/0/0)
20:1	1 (0/0/1)	0 (0/0/0)	1 (0/0/1)	1 (1/0/0)	1 (0/0/1)	2 (1/1/0)
20:2	1 (0/0/1)	2 (0/0/2)	1 (0/0/1)	2 (0/0/2)	1 (0/0/1)	3 (0/1/2)
20:3	2 (1/0/1)	3 (0/1/2)	4 (1/1/2)	4 (0/1/3)	3 (1/0/2)	3 (0/0/3)
20:4	0 (0/0/0)	11 (0/1/10)	1 (0/0/1)	15 (1/2/12)	4 (0/1/3)	9 (0/1/8)
20:5	2 (1/1/0)	2 (0/1/1)	1 (1/0/0)	2 (0/1/1)	2 (1/1/0)	2 (0/1/1)
22:0	0 (0/0/0)	1 (1/0/0)	0 (0/0/0)	1 (1/0/0)	0 (0/0/0)	1 (1/0/0)
22:1	0 (0/0/0)	1 (0/1/0)	0 (0/0/0)	0 (0/0/0)	0 (0/0/0)	1 (0/1/0)
22:4	1 (0/0/1)	1 (1/0/0)	1 (0/0/1)	1 (1/0/0)	2 (0/0/2)	1 (1/0/0)
22:5	2 (0/1/1)	3 (0/0/3)	2 (0/0/2)	3 (0/0/3)	3 (0/0/3)	3 (0/0/3)
22:6	2 (0/0/2)	5 (0/0/5)	3 (0/0/3)	5 (0/0/5)	4 (0/0/4)	6 (0/1/05)
GpIns	13 (0/4/9)	2 (0/0/2)	12 (0/3/9)	4 (0/1/3)	22 (0/5/17)	0 (0/0/0)
GpEtn	1 (0/0/1)	12 (0/2/10)	0 (0/0/0)	20 (0/3/17)	0 (0/0/0)	22 (0/5/17)
GpCho	4 (0/1/3)	9 (0/1/8)	5 (0/1/4)	9 (0/0/9)	3 (0/0/3)	12 (0/2/10)
GpGro	6 (0/0/6)	2 (0/0/2)	5 (0/0/5)	2 (0/0/2)	1 (0/0/1)	2 (0/0/2)
GpSer	0 (0/0/0)	2 (0/0/2)	0 (0/0/0)	2 (0/0/2)	0 (0/0/0)	2 (0/0/2)
GPA	5 (0/5/0)	3 (0/2/1)	0 (0/0/0)	4 (0/3/1)	6 (0/5/1)	5 (0/5/0)

Values are given as: total (fatty acid/monoacylphospholipids/diacylphospholipids). For example, at day 14 eighteen species containing 16:0 were decreased with increasing dose of PB and this can be broken down into the free fatty acid, 3 monoacylphospholipids and 14 diacylphospholipids (1/3/14).

Direct infusion ESI-MS showed that species containing stearic acid (18:0) and palmitic acid (16:0) were generally increased by exposure to PB. The exceptions were if they were in combination with a highly unsaturated fatty acid (HUFA), especially 20:4 (arachidonic acid), docosahexaenoic acid 22:6 (docosahexaenoic acid) or 22:5, when they tended to be decreased (Additional file 3. The class of phospholipid is also important, with large changes in the proportions of different phospholipid classes present in the liver being observed. There was a general increase in GpIns species at all time points (Figure 7). The only GpIns species that were decreased were those containing arachidonic acid (20:4). Most GpCho and GpEtn lipids were decreased across all time points. Those GpCho and GpEtn species which increased all contained stearic acid (18:0) (Additional file 3).

## Discussion

In this study we have examined the well characterized non genotoxic carcinogen PB to investigate whether a combined transcriptomic and metabolomic approach could identify early stage mechanistic markers associated with PB toxicity. To confirm that PB was producing the effects reported previously, we measured a number of classical parameters. Dietary administration of PB resulted in an increase in relative liver weight (table 1), centrilobular hypertrophy (figure 1) and a transient increase in cell replication (table 1), all of which are in accordance with the literature [3,4,9]. Both metabolomics and transcriptomics identified changes in liver metabolism after just one day of exposure to PB demonstrating the sensitivity of this approach. Many metabolites were identified as having altered in both liver and plasma and in order to aid interpretation of these results, metabolite and transcript data were integrated highlighting several pathways which had been perturbed by PB.

### Hepatic glycogen

A large, dose dependent decrease in the amount of glycogen in the livers of the animals given PB was observed at all time points. Both direct mapping of changes in gene expression onto the pathways and PLS regression between transcriptional and metabolomic data identified a decrease in glucokinase as being correlated with the decrease in glycogen. Glucokinase catalyses the phosphorylation of glucose to glucose-6-phosphate and is involved in controlling the rate of glycogen synthesis and glycolysis [14]. This indicates that the decrease in glycogen detected in the livers of animals exposed to PB is a result of a decrease in glycogen synthesis, with this previously being reported *in vitro *in isolated rat hepatocytes [15,16].

### Hepatic glutathione

Hepatic glutathione was increased at days 1, 3 and 7 in animals exposed to PB relative to control animals. This was accompanied by an increase in the expression of glutamate-cysteine ligase, the rate limiting enzyme in the synthesis of glutathione, at days 1 and 3 in animals exposed to PB. There are mixed reports as to whether PB exposure increases glutathione [17-19], but in this study the combined transcriptomic and metabolomic data indicates PB increased hepatic glutathione by an increase in synthesis. The majority of γ-glutamylcysteine is converted to glutathione by glutathione synthase, but a small amount is converted to 5-oxoproline and cysteine by γ-glutamylcyclotransferase [20] (figure 5). The proportions of γ-glutamylcysteine being metabolized by each path is controlled by the kinetics of the two enzymes, and thus, the detected increase in 5-oxoproline with PB dose suggests that flux through both pathways has increased.

Glutathione reductase expression was increased for animals in the mid and high dose groups at all time points. This enzyme reduces oxidized glutathione back to reduced glutathione in a NADPH dependent reaction and provides an important anti-oxidative stress mechanism in the liver. A number of mechanisms generate NADPH for this conversion, including the conversion of malate to pyruvate catalyzed by malic enzyme. The expression of malic enzyme is highly correlated with glutathione reductase expression (Pearson correlation coefficient, r = 0.7), suggesting that the increase in malic enzyme expression is due to increased demand for reducing power which is associated with drug metabolism and PB exposure [21].

### Hepatic succinate

The increase in hepatic succinate caused by exposure to PB could have been caused by alterations in a number of different pathways, including increased TCA rate and increased reducing potential demand by the liver cells. However, using our combined metabolomic and transcriptomic approach we demonstrate that succinate is correlated with the increased expression of ALAS. This enzyme catalyses the reaction between glycine and succinyl CoA to form 5-aminolevulinate, in the first committed step in heme biosynthesis [22] and PB induces ALAS in isolated hepatocytes [23]. PB induces the CYP2B family of cytochrome P450 enzymes and hence the increase in heme biosynthesis induced by PB is consistent with the heme requirements associated with induction of cytochrome P450s [24].

### Plasma lipids

Clinical biochemistry measurements showed a decrease in plasma triglycerides in animals given the mid and high doses of PB at day 14 and ^1^H-NMR of plasma also showed a decrease in lipid resonances at days 7 and 14. PLS demonstrated a correlation between the CH_2 _resonance of plasma lipoproteins and LPL expression. LPL is not normally expressed in liver tissue of adult rats. However, it is expressed in the livers of neonatal rats and its expression can be induced in adult rats, for example by the PPAR agonist fenofibrate [25,26]. LPL is found in the endothelial cells lining capillaries of tissues and hydrolyses triglycerides from lipoproteins circulating in the plasma to release fatty acids which can be taken up by the tissue. Its induction in the liver of rats exposed to PB may be responsible for the decrease in plasma lipids, suggesting that the liver in these animals has an increased demand for fatty acids, possibly due to the proliferation in endoplasmic reticulum which is known to be induced by PB [4,27,28].

High resolution ^1^H-NMR spectroscopic analysis of plasma demonstrated differences in the lipoprotein fraction with increasing PB dose. There is a subtle decrease in chemical shift as the density of the lipoproteins decreases and this affects the shape of the lipid peaks detected in blood plasma [13]. At day 14, spectra from rats exposed to PB demonstrated a shift in the terminal methyl lipoprotein resonance to a lower chemical shift reflecting an increase in high density lipoprotein and decrease in low density lipoprotein as has been reported in PB-exposed Sprague Dawley rats [29].

### Lipid remodeling in the liver

The decrease in glycogen and increase in fatty acid uptake in response to PB suggested that the proportions of fat and carbohydrate in the liver were altered. HRMAS ^1^H-NMR spectroscopy was used to study liver tissue from day 14 and demonstrated a clear increase in the ratio of lipid to carbohydrate in rats exposed to 500 and 1000 ppm PB. ^1^H-NMR showed a large, dose dependent decrease in the levels of hepatic glucose and glycogen at all time points. Expression of lipoprotein lipase was increased in the high dose groups from day 3, and this is also the time point at which changes in the lipid species present were first detected by GC-MS. Because of the relative low spinning speeds used during HRMAS ^1^H NMR spectroscopy, the technique focuses on the detection of lipids with some mobility, and hence the increased lipid detected in the spectra reflect the mobilization of triglyceride stores within the liver tissue.

Direct infusion mass spectrometry provided an important tool for rationalising the total lipid changes detected by GC-MS and HRMAS ^1^H NMR spectroscopy. The lipid changes were characterised by a global increase in GpIns apart from those containing HUFAs, while most GpCho and GpEtn species were decreased. The synthesis of glycerophospholipids is complex. GpIns and GpSer are both synthesized from CDP-DAG, while GpEtn is synthesized from the decarboxylation of GpSer. GpCho is synthesized either from GpSer by sequential methylation by phosphatidylethanolamine *N*-methyltransferase and *S*-adenosylmethionine or direct from CDPcholine and DAG via CDP-choline:1,2-diacylglycerol cholinephosphotransferase. In addition CDPethanolamine and DAG are also precursors to GpEth. In mammals this remodelling of glycerophopsholipids largely takes place in the ER [for a discussion of these pathways see http://www.lipidlibrary.co.uk/Lipids/complex.html]. PB induces proliferation of the ER, and while the key enzymes involved in the synthesis of GpIns, GpEtn and GpCho are all found within the ER, in the present study PB selectively increased the concentration of GpIns, while other phospholipids largely decreased in concentration. This suggests PB exposure induces a remodelling of cellular lipids, which may in turn relate to the biochemical changes associated with pathological ER proliferation and the need to synthesize particular phospholipids for membrane synthesis.

### Omic techniques

In this study the use of metabolomics and transcriptomics has allowed the detection of many changes occurring in response to PB using a small set of experiments and without predefined hypotheses. This is the first time that metabolomics and transcriptomics have been used in combination to study the effects of PB exposure and the published data available has been used to validate the approach. This ability of omic techniques to identify changes occurring across many metabolic pathways and in different tissues and biofluids, without the need for a large number of targeted analyses, is one of the technology's key benefits and is well demonstrated in this work. In addition, the transcriptomic data strengthened the metabolomic data by improving the interpretability of the metabolic changes. It was possible to determine if a decrease in synthesis or an increase in degradation was the cause of the decrease in the concentration of a metabolite (as in hepatic glycogen) and the cause of changes in metabolites which lie on many pathways were also identified (as in hepatic succinate). The transcriptomic data is enhanced by metabolomic data as changes in gene expression do not necessarily imply changes in levels of enzymes and metabolite and as not all proteins are under transcriptional control, gene expression profiling can miss large changes which can be detected with metabolomics [30,31].

## Conclusions

In this study we have used a combined omic platform to investigate early stage mechanistic biomarkers associated with exposure to the classic rodent non-genotoxic carcinogen PB. The use of metabolomics and transcriptomics in combination greatly enhanced the conclusions which could be drawn from the dataset by aiding the interpretation of metabolomic data and by linking changes in gene expression to measured changes in metabolism. Data available on this compound has allowed us to confirm our findings and interpretation, validating the use of this approach for the study of less well characterized compounds. Pathway perturbations included those associated with glycogen synthesis, glycolysis, glutathione metabolism and heme synthesis. Furthermore, the approach was capable of identifying diverse mechanistic responses, even for metabolites like succinate which form a central hub in metabolism where a number of pathways converge. This study has shown the sensitivity of 'omic technologies to identify many perturbations without a predefined hypothesis, providing early stage markers of the subsequent pathophysiology.

## Methods

### Animal handling and sample collection

All animal procedures conformed to the Home Office (UK) guidelines for experimentation with animals and were approved by local ethics committee at Syngenta. Eighty nine week old male Fischer (F344) rats were randomly divided into four groups of twenty animals (Additional file 4). Each group was exposed to a different concentration (0, 50, 500 or 1000 ppm) of the sodium salt of phenobarbital which was added to the standard laboratory diet and milled to homogeneity. Animals were fed ad lib throughout the study. Five animals from each group were killed by exsanguination under halothane anesthesia after 1, 3, 7 and 14 days of PB exposure. Blood was collected into lithium heparin tubes, centrifuged at 2000 rpm for 10 minutes and the plasma separated for clinical biochemistry. Plasma samples for metabolomic analysis were stored at -80°C until analysis. Livers were removed immediately and weighed. Samples of liver (~150 mg) were taken from the left lateral lobe and snap frozen in liquid nitrogen for genomic and metabolomic analysis. Representative samples of liver for histopathology were taken from the three main lobes, fixed for 48 hours in 10% neutral buffered formol saline and processed to paraffin wax blocks. Sections of liver (4 μm) were stained for ki67 by immunohistochemistry and the labeled nuclei counted to produce a labeling index. A sample of liver was taken from the right median lobe, fixed in 3% glutaraldehyde in cacodylate buffer, processed, embedded and examined by electron microscopy.

### Materials

All chemicals were purchased from Sigma-Aldrich (Poole, UK) with the exception of D_2_O, sodium-3-(trimethylsilyl)-2,2,3,3-tetradeuteriopropionate (TSP) and N-Methyl-N-(trimethylsilyl)trifluoroacetamide (MSTFA) which were obtained from Goss Scientific (Great Baddow, UK), Cambridge Isotope Laboratories (Andover, MA) and Macherey-Nagel (Düren, Germany), respectively.

### Clinical chemistry

Plasma was analysed for the following parameters: total protein, total bilirubin, creatine kinase activity, aspartate aminotransferase activity, cholesterol, creatinine, albumin, alkaline phosphatase activity, gamma-glutamyl transferase activity, triglycerides and glutamate dehydrogenase using a Konelab clinical chemistry analyzer (Thermo Scientific, Waltham, MA).

### Tissue extraction

Tissue samples were extracted using methanol-chloroform as described by Le Belle et al [32]. Approximately 75 mg of frozen tissue was ground up with dry ice. Methanol-chloroform solution (2:1, 600 μl) was added and the samples were sonicated for 15 min. Chloroform-water (1:1, 400 μl) was added and the samples centrifuged (20 min, 13 500 rpm). Aliquots of the resulting aqueous and organic layers were taken for GC-MS, LC-MS and ^1^H-NMR analysis. The aqueous fractions were dried in an evacuated centrifuge. The organic fractions and pellets were dried in a fume hood overnight.

### High Resolution ^1^H-NMR spectroscopy

For analysis of liver tissue extracts, dried aqueous extracts from 500 μl of the aqueous layer were dissolved in phosphate buffered D_2_O (pH 7.0, 600 μl, 33 mM Na_2_HPO_4_, 6.7 mM NaH_2_PO_4_, 0.1% sodium azide, 0.17 mM TSP). ^1^H-NMR spectra were acquired on a Bruker DPX400 spectrometer operating at 400.13 Hz using a 5 mm probe. The standard "noesypr1d" pulse sequence (Bruker Analytik GmbH, Germany) was used for solvent suppression (relaxation delay = 2 s, t1 = 3 μs, mixing time = 150 ms, solvent presaturation applied during the relaxation time and the mixing time). 128 transients were collected into 16 K data points over a spectral width of 12 ppm at 37°C. NMR free induction decays (FIDs) were Fourier transformed with a line broadening of 0.3 Hz. The spectra were phased, base line corrected, referenced to TSP at 0.00 pm and integrated using ACD labs NMR Processor (version 8, ACD, Toronto, Canada). The spectra were integrated in 0.01 ppm buckets between 0.20 and 9.95 ppm excluding the water region (4.72-5.05 ppm). To account for concentration differences, the total integral of each spectrum was normalized to 10000 and each peak represented as a ratio to the normalized total integral. However, it became apparent that there were very large differences in the amount of glucose and glycogen in the liver extract samples and so the spectra were also renormalized with the glucose and glycogen resonances (3.35-4.05 ppm, 4.60-4.72 ppm, 5.20-5.55 ppm) excluded (Additional files 5).

For plasma analysis, plasma (250 μl) was added to 500 μl of deuterated acetonitrile/D_2_O (50:50). Samples were mixed and centrifuged (12 000 rpm, 5 minutes) and 600 μl of supernatant taken for analysis. ^1^H-NMR spectra were acquired on a Bruker DPX400 spectrometer operating at 400.13 Hz using a 5 mm probe. Samples were analysed using a Carr-Purcell-Meiboom-Gill (CPMG) pulse sequence to suppress the contribution from large molecules which also included a water pre-saturation regime. 128 transients were collected into 16 k data points across a spectral width of 20 ppm at 37°C. The relaxation delay was 2 s and the spin echo delay (d20) was 1 ms. 48 loops (spin echoes) were used to give a total spin echo time of 96 ms. NMR FIDs were Fourier transformed with a line broadening of 0.3 Hz. The spectra were phased, base line corrected, referenced to the glucose doublet at 5.23 ppm and integrated using ACD labs NMR Processor (version 8, ACD, Toronto, Canada). The spectra were integrated in 0.02 ppm buckets between 0.60 and 8.00 ppm excluding the water region (4.68-5.15 ppm). To account for concentration differences, the total integral of each spectrum was normalized to 10000 and each peak represented as a ratio to the normalized total integral.

For HRMAS ^1^H NMR experiments approximately 15 mg of tissue was soaked in D_2_O with 10 mM TSP then placed in a 4 mm diameter zirconium oxide rotor for analysis. The sample was packed into a homogeneous ball of 30 μl volume using a Teflon spacer. HRMAS experiments were conducted on a Bruker AVANCE II+ spectrometer operating at 500.13 MHz for the ^1^H frequency and using a high resolution 4 mm MAS probe. Samples were spun at a frequency of 4500 Hz at a temperature of 27°C. A "noesypr1d" pulse sequence was used (relaxation delay = 2.5 s, t1 = 4 μs, mixing time = 50 ms, solvent presaturation applied during the relaxation time and the mixing time). 64 transients were collected into 32 k data points across a spectral width of 16 ppm. FIDs were Fourier transformed with a line broadening of 1 Hz The spectra were phased, base line corrected, referenced to TSP at 0.00 pm and integrated using ACD labs NMR Processor (version 8, ACD, Toronto, Canada). The spectra were integrated in 0.04 ppm buckets between 0.50 and 5.60 ppm, excluding the water region (4.75-5.00 ppm).

### Gas Chromatography Mass Spectrometry (GC-MS)

The dried aqueous extracts from 100 μl of the aqueous phase of the extraction mixture were derivatised with methoxyamine hydrochloride in pyridine (20 mg ml^-1^, 30 μl, 17 hr, 22°C) and silylated using MSTFA (30 μl, 1 hr, 22°C) [33]. 50 μl of the derivatised sample was added to 200 μl hexane prior to analysis (Additional files 5).

The dried organic fraction was treated with a methylating reagent which formed the fatty acid methyl esters of carboxylic acids. Chloroform-methanol (1:1, 750 μl) and boron trifluoride in methanol (~10%, 125 μl) were added to the extract and the samples heated to 80°C for 90 minutes. Deionized water (300 μl) and hexane (600 μl) were added, the samples vortex mixed and the lower layer was removed and dried. The dried samples were reconstituted in 150 μl hexane prior to analysis [34] (Additional files 5).

GC-MS analysis was carried out using a Thermo Electron Trace GC Ultra coupled to a Thermo Electron DSQ single quadrupole mass spectrometer. For all analyses the injector temperature was 230°C. Helium was used as the carrier gas and maintained at a constant flow of 1.2 ml min^-1^. The MS was operated in full scan mode with 3 scans s^-1 ^across a range of 50-650 m/z.

Derivatised aqueous extracts were analysed using a 30 m × 0.25 mm ID × 0.25 μm df 5%-phenyl-95%-dimethylpolysiloxane column. 1 μl derivatised sample was injected splitless. The initial column temperature of 70°C was held for 2 minutes then the temperature was ramped at 5°C min^-1 ^to 310°C and this temperature was held for 20 minutes.

Derivatised organic extracts were analysed on a 30 m × 0.25 mm × 0.25 μm 100% polyethylene glycol column. 2 μl derivatised sample was injected with a split ratio of 20:1. The initial column temperature of 60°C was held for 2 min and then the temperature was ramped at 6°C min^-1 ^to 230°C and this temperature was held for 7 min. GC-MS data was analyzed using Qual Browser version 1.4 (Thermo Electron Corporation). Peaks were integrated individually and the total integrated peak area of each spectrum normalized to 10000.

### Univariate statistical analysis

Liver and body weight, clinical chemistry, food consumption and Ki67 staining were analysed by 1-way analysis of variance to identify differences between the dose groups. Dunnett's multiple comparison test was then used to identify which of the treatment group means were significantly different to the control group mean [35].

### Multivariate statistical analysis

Normalized data was imported into SIMCA-P+ (v. 11, Umetrics, Umeå, Sweden) for multivariate statistical analysis. The data was mean centered and scaled. Scaling is necessary so that high concentration metabolites do not dominate the models. NMR data was Pareto scaled to suppress the contribution from regions of the spectra containing no peaks. Since in the GC-MS analysis only identified peaks were used, GC-MS data was scaled to unit variance. Initially, PCA models were built to look for clustering and identify outliers, and then PLS was applied to improve the separation and look for changes correlated with increasing dose of PB. These are essentially data reduction methods whereby the major variation in the dataset is summarized in new variables known as components. Thus, a dataset which has thousands of variable can be summarized in typically fewer than ten new components [36]. PCA is an unsupervised technique which identifies the major sources of variation in the dataset. PLS is a supervised technique which identifies variation in the dataset (the X-block) which correlates with a specific variable (the Y-variable), in this study the dose of PB. R^2 ^and Q^2 ^values were used to assess the amount of variation represented by the principal components and robustness of the model, respectively. To prevent over fitting, the predictive capability of the models was checked by samples from the dataset, building a model and then predicting the dose for the omitted samples. The validate function in SIMCA was also used to check the models. This function randomly permutes the Y variables and calculates the R^2 ^and Q^2^. As the similarity between the actual doses and the permuted doses falls, so should the R^2 ^and Q^2 ^values.

### Gene expression analysis

Total RNA was isolated from 150 mg of tissue using a midi column according to the manufactures instructions (Qiagen; West Sussex, UK). RNA quality and concentration were determined using an Agilent Bioanalyzer and samples were repurified if the calculated RNA Integrity Number was below 7. Purified RNA was converted to complementary DNA, amplified and then converted to biotin-labeled complementary RNA using Affymetrix GeneChip 3' Amplification reagents (Affymetrix, High Wycombe). 15 ug was hybridized to Affymetrix Rat Expression RG-230 v2 GeneChips as described in the Affymetrix GeneChip Expression Analysis Technical Manual http://www.affymetrix.com/support/technical/manual/expression.manual.affx. The probe arrays were scanned and the probeset intensities were combined and averaged using Microarray Suite 5.0 (Affymetrix). The mean of each array was globally normalized to 500. Data were imported into GeneSpring GX version 7 and the following normalizations performed: data transformation to set expression values below 0.01 to 0.01; per gene normalization, where the signal strength of each gene is normalized to the median of its signal strength in all samples. The microarray data is available at Gene Expression Omnibus (GSE18753).

The probesets on the array were then analyzed by a number of filters and statistical tests. Firstly to remove probesets for genes without detectable expression: genes that did not have at least 3 present flags were excluded and genes that had an expression value of less than 90 in at least 3 samples were also excluded. To ensure robust detection of any differentially expressed genes any probeset that showed less than a 1.5-fold change in expression relative to its time-matched control were also excluded. To detect significant changes in expression levels a 2-way analysis of variance was performed on the filtered gene list (using a Benjamini and Hochberg false discovery rate of < 0.05) on time and treatment. Ratios of changes in gene expression were calculated by normalizing each treated sample to the median value of the time-matched control. These ratios were used for hierarchical clustering of the differentially expressed genes using the Pearson correlation coefficient. Differentially expressed genes were functionally categorized using their Gene Ontology (GO) and Kyoto Encyclopedia of Genes and Genomes (KEGG) pathways annotations using GeneSpring GX. GO over-representation analysis was performed using the GeneSpring GX GO browser. The significance of a Gene Ontology Biological Process being over-represented in a gene list relative to the annotations on the chip was assessed using a hypergeometric p-value < 0.05 without multiple testing corrections. KEGG pathway over-representation was performed using the GeneSpring GX script "SG3b-1 Find genes in a GL present in PATHWAYS" contained within the Bioscript Library 2.2 (Agilent). A pathway needed to have at least 3 differentially expressed genes and a p < 0.05 to be included in the analysis [37].

### Integration of metabolomic and genomic data

Two approaches were taken to integrate the data generated by metabolomic and genomic analyses. Firstly, significant changes in gene expression were mapped on to Kyoto Encyclopedia of Genes and Genomes (KEGG) metabolic pathways using GeneSpring (Agilent Technologies, Santa Clara, CA). This allowed the identification of pathways where both the concentrations of transcripts and metabolites were altered.

In the second approach, PLS was used to build regression models using the genomic data as the X-block and the normalized integrated areas of peaks corresponding to individual metabolites identified which were altered by PB exposure as the Y-variable. PLS is an extension of PCA where a projection model is developed predicting Y from X via scores of X, and is a generalized multiple regression method suitable for multiple collinear X and Y variables. This method identifies changes in the gene expression data which correlate with changes in the concentration of a particular metabolite and is particularly advantageous when investigating changes in metabolites which lie on many pathways. Correlations were identified by the examination of the X and Y components of the first component of the PLS model. In addition, the goodness of fit (Q^2^) of the model was evaluated by excluding every 7^th ^sample as part of a leave one out cross validation process. Q^2 ^is defined as the fraction of the total variation of the X matrix that can be predicted by a component as estimated by cross-validation and Q^2 ^= (1.0-PRESS/SS) where PRESS is the predicted error sum of squares which is the squared difference between observed Y and predicted Y and SS is the residual sum of squares of the previous component.

The loading plots for the PLS model show the correlation between the X variables, or their residuals in subsequent dimensions, in the first dimension, and the Y variables (or Y residuals scores in subsequent dimensions). The weights are selected so as to maximize the covariance between the X matrix and Y block. The loadings were ranked according to magnitude and in this way, the transcripts which were most highly correlated with changes in the concentration of a particular metabolite could be identified. Correlates were considered as significant if Q^2^>0.40 and the procedure passed the cross-validation routine in Simca-P+ whereby the real Q^2 ^was calculated to be greater than 30 models generated using a random permutation of the Y scores.

## Abbreviations

ALAS: 5-aminolevulinate synthase; GC-MS: gas chromatography-mass spectrometry; LPL: lipoprotein lipase; MAS: magic angle spinning; MSTFA: N-Methyl-N-(trimethylsilyl)trifluoroacetamide; NMR: nuclear magnetic resonance spectrometry; PB: phenobarbital; PCA: principal components analysis; PLS: partial least squares; TSP: sodium-3-(trimethylsilyl)-2,2,3,3-tetradeuteriopropionate.

## Authors' contributions

CLW and JLG performed all the metabolomics analyses, apart from the biofluid analysis which was performed with LAC. Transcriptomic analysis was performed by RAC, and JD managed the animal study. CLW, RAC and JLG carried out the pattern recognition in the paper. JW performed and interpreted histology data, and the study was designed and conceived by JD, JW and CJW. CLW drafted the paper under the supervision of CJW and JLG. All authors read and approved the final manuscript.

## Supplementary Material

Additional file 1 **Metabolite changes in response to increasing dose of phenobarbital**. Metabolite changes in liver and plasma in response to increasing dose of PB as detected by ^1^H-NMR spectroscopy and GC-MS across the time course of the study. Click here for file

Additional file 2 **Pathways identified as perturbed by transcriptomic analysis**. Pathways and biological processes which are over represented in the set of genes whose expression is altered by phenobarbital. Genes are grouped by temporal expression profile as described Figure 4. Click here for file

Additional file 3 **Remodelling of the lipid profile of liver tissue following PB treatment**. Changes in intact phospholipids in response to increasing dose of PB as detected by negative mode direct infusion ESI-MS of extracts from liver tissue. Click here for file

Additional file 4 **Study design for animal experiment**. This table depicts the animal numbers used in the design of the study. Click here for file

Additional file 5 **Metabolomics Standards Initiative Compliant Metadata**. The excel spread sheets contain all the metabolomics data collected by NMR spectroscopy and GC-MS of fatty acid methyl esters according to the Standard reporting requirements for biological samples in metabolomics experiments: mammalian/in vivo experiments. Click here for file
