# Supplementary material for: An integrated functional genomic study of acute phenobarbital exposure in the rat
Source: BMC Genomics. 2010 Jan 6;11:9. doi: 10.1186/1471-2164-11-9 (PMC2826316; doi:10.1186/1471-2164-11-9)
Supplement: Additional file 1 — Metabolite changes in response to increasing dose of phenobarbital. Metabolite changes in liver and plasma in response to increasing dose of PB as detected by 1H-NMR spectroscopy and GC-MS across the time course of the study. [file 1471-2164-11-9-S1.DOCX]

## Additional file 1 - Metabolite changes in response to increasing dose of phenobarbital

| **Platform** | **Time Point** | **Relative increase with increasing dose of PB** | **Relative decrease with increasing dose of PB** |
| --- | --- | --- | --- |
| **^1^H-NMR analysis of liver extract aqueous fraction** | Day 1 | succinate  glutamine  glutathione (slight) | Glucose  glycogen  adenosine  leucine |
|  | Day 3 | succinate  glutamine  glutathione  taurine | Leucine  valine  choline  glucose  glycogen  adenosine |
|  | Day 7 | succinate  glutamine  glutathione  taurine | Glucose  glycogen  adenosine |
|  | Day 14 | succinate  phosphocholine | Glucose  glycogen  adenosine  nicotinamide  adenosine |
| **GC-MS analysis of liver extract aqueous fraction** | Day 1 | succinate  5-oxoproline  beta hydroxybutyrate  threonate  inositol isomer  citrate  glyceric acid  glycine | Disaccharides  ethanolamine  fructose |
|  | Day 3 | alanine  lactate  5-oxoproline  glycine  succinate  glutamate  threonate | Disaccharides  ethanolamine  fructose  ribose  phenylalanine  tyrosine |
|  | Day 7 | citrate  lysine  threonate  succinate  ornithine  ethanolamine phosphate  fumarate  inositol isomer  glycine | Disaccharides  fructose  ethanolamine |
|  | Day 14 | glycine  serine  threonine  ornithine  isoleucine  5-oxoproline  succinate  proline  alanine  threonate | Ribose  malate |
| **GC-MS analysis of liver extract lipid fraction** | Day 1 | - | - |
|  | Day 3 | 16:1  9-18:1 | 18:0 (branched chain)  19:0 isomer  5,8,11,14-20:4  4,7,10,13,16,19-22:6  18:1  16:0 (branched chain)  15:0 |
|  | Day 7 | 5,8,11,14,17-20:5  9-18:1  16:1  8,11,14-20:3 | 10:0  15:0  11,14-20:2  9,12,15-18:3  4,7,10,13,16,19-22:6  12:0  5,8,11,14-20:4 |
|  | Day 14 | 18:1  9-18:1  5,8,11,14,17-20:5 | 14:0  16:0  17:0  18:0 (branched chain)  11-20:1 |
| **^1^H-NMR analysis of plasma** | Day 1 | - | - |
|  | Day 3 | -CH_3_ lipid (0.84-0.86)  branched chain amino acids (slight)  lactate  alanine  glutamate  glutamine | -CH_2_- lipid (slight) |
|  | Day 7 | branched chain amino acids  lactate  acetate (slight)  glutamate  glutamine  choline/ phosphocholine | -CH_2_- lipid  -CH_2_CH_2_CO_2_H  -CH=CHCH_2_CH=CH-  -CH_2_CH=CHCH_2_- |
|  | Day 14 | -CH_3_ lipid (0.84-0.86)  branched chain amino acids  lactate  alanine (slight)  acetate  glutamate  glutamine  choline/ phosphocholine | -CH_3_ lipid (0.88-0.90)  -CH_2_- lipid  -CH_2_CH_2_CO_2_H  -CH_2_CH_2_CO_2_H  -CH=CHCH_2_CH=CH-  -CH_2_CH=CHCH_2_- |

Metabolite changes in liver and plasma in response to increasing dose of PB as detected by ^1^H-NMR spectroscopy and GC-MS across the time course of the study.
