# Supplementary material for: An integrated functional genomic study of acute phenobarbital exposure in the rat
Source: BMC Genomics. 2010 Jan 6;11:9. doi: 10.1186/1471-2164-11-9 (PMC2826316; doi:10.1186/1471-2164-11-9)
Supplement: Additional file 2 — Pathways identified as perturbed by transcriptomic analysis. Pathways and biological processes which are over represented in the set of genes whose expression is altered by phenobarbital. Genes are grouped by temporal expression profile as described Figure 4. [file 1471-2164-11-9-S2.DOCX]

## Additional file 2 - Pathways identified as perturbed by transcriptomic analysis

|  | GO Biological Process | p-value | GO Cellular Component | p-value | KEGG or GenMAPP pathway | p-value |
| --- | --- | --- | --- | --- | --- | --- |
| Transient genes | cell cycle | 4E-10 | Nucleus | 9E-06 | cell cycle | 3E-12 |
|  | mitosis | 4E-07 | Cytoplasm | 2E-02 | calcium signaling pathway | 0.0202 |
|  | regulation of cell cycle | 1E-06 |  |  | Pantothenate and CoA biosynthesis | 0.0003 |
|  | Cytokinesis | 2E-04 |  |  | starch and sucrose metabolism | 0.001 |
|  | proteolysis and peptidolysis | 1E-02 |  |  | tryptophan metabolism | 0.004 |
|  | protein amino acid phosphorylation | 1E-02 |  |  | proteasome | 0.0007 |
|  | regulation of transcription, DNA-dependent | 3E-02 |  |  | Proteasome degradation | 0.0009 |
|  | electron transport | 1E-02 |  |  |  |  |
|  | regulation of transcription, DNA-dependent | 2E-02 |  |  |  |  |
|  | electron transport | 1E-02 |  |  |  |  |
| Sustained down regulation | neurotransmitter transport | 3E-04 | integral to plasma membrane | 4E-05 | alanine and aspartate metabolism | 1E-03 |
|  | electron transport | 7E-03 | Microsome | 5E-05 | glutamate metabolism | 2E-03 |
|  | Metabolism | 4E-02 | Peroxisome | 2E-04 | urea cycle and metabolism of amino groups | 2E-03 |
|  |  |  | integral to membrane | 1E-03 | glycine, serine and threonine metabolism | 4E-03 |
|  |  |  | Mitochondrion | 2E-03 | arginine and proline metabolism | 6E-03 |
|  |  |  | endoplasmic reticulum | 5E-03 | glycolysis and gluconeogenesis | 6E-03 |
|  |  |  | Membrane | 9E-03 | tight junction | 2E-02 |
| Sustained upregulation | xenobiotic metabolism | 2E-04 | Membrane | 4E-03 | gamma-Hexachlorocyclohexane degradation | 4E-06 |
|  | induction of apoptosis | 3E-03 | Microsome | 7E-03 | glutathione metabolism | 4E-05 |
|  | signal transduction | 6E-03 | endoplasmic reticulum | 2E-02 | pentose and glucuronate interconversions | 0.0002 |
|  | Metabolism | 2E-02 |  |  | porphyrin and chlorophyll metabolism | 0.0005 |
|  | electron transport | 2E-02 |  |  | alzheimer's disease | 0.001 |
|  | proteolysis and peptidolysis | 3E-02 |  |  | oxidative_Stress | 0.001 |
|  |  |  |  |  | starch and sucrose metabolism | 0.002 |
|  |  |  |  |  | fatty acid metabolism | 0.003 |
|  |  |  |  |  | androgen and estrogen metabolism | 0.003 |
|  |  |  |  |  | tryptophan metabolism | 0.004 |
|  |  |  |  |  | purine metabolism | 0.02 |
|  |  |  |  |  | regulation of actin cytoskeleton | 0.05 |

Pathways and biological processes which are over represented in the set of genes whose expression is altered by phenobarbital. Genes are grouped by temporal expression profile as described Figure 4.
