# Supplementary material for: An integrated functional genomic study of acute phenobarbital exposure in the rat
Source: BMC Genomics. 2010 Jan 6;11:9. doi: 10.1186/1471-2164-11-9 (PMC2826316; doi:10.1186/1471-2164-11-9)

**Additional file 3:** Changes in intact phospholipids in response to increasing dose of PB as detected by negative mode direct infusion ESI-MS of extracts from liver tissue.


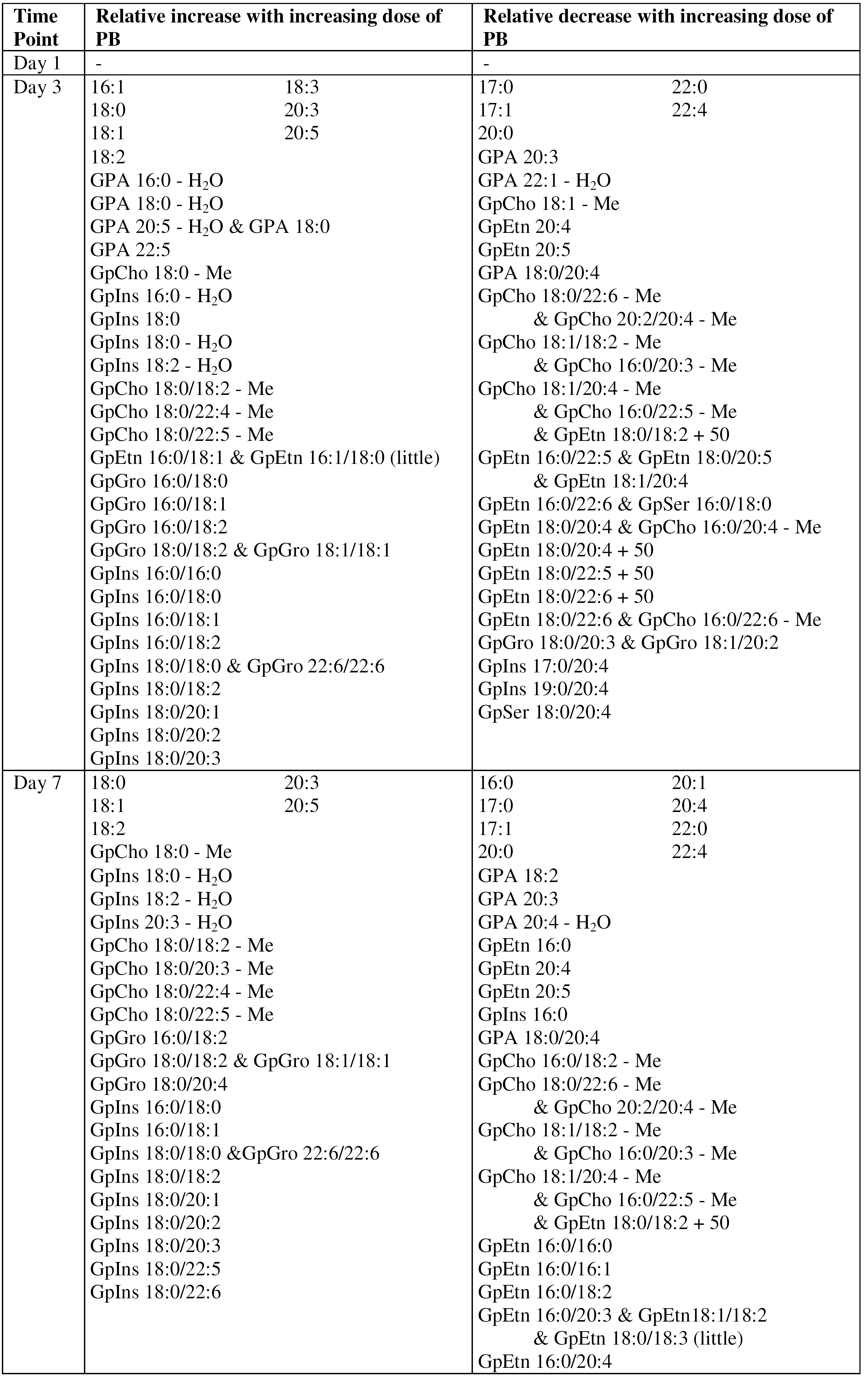


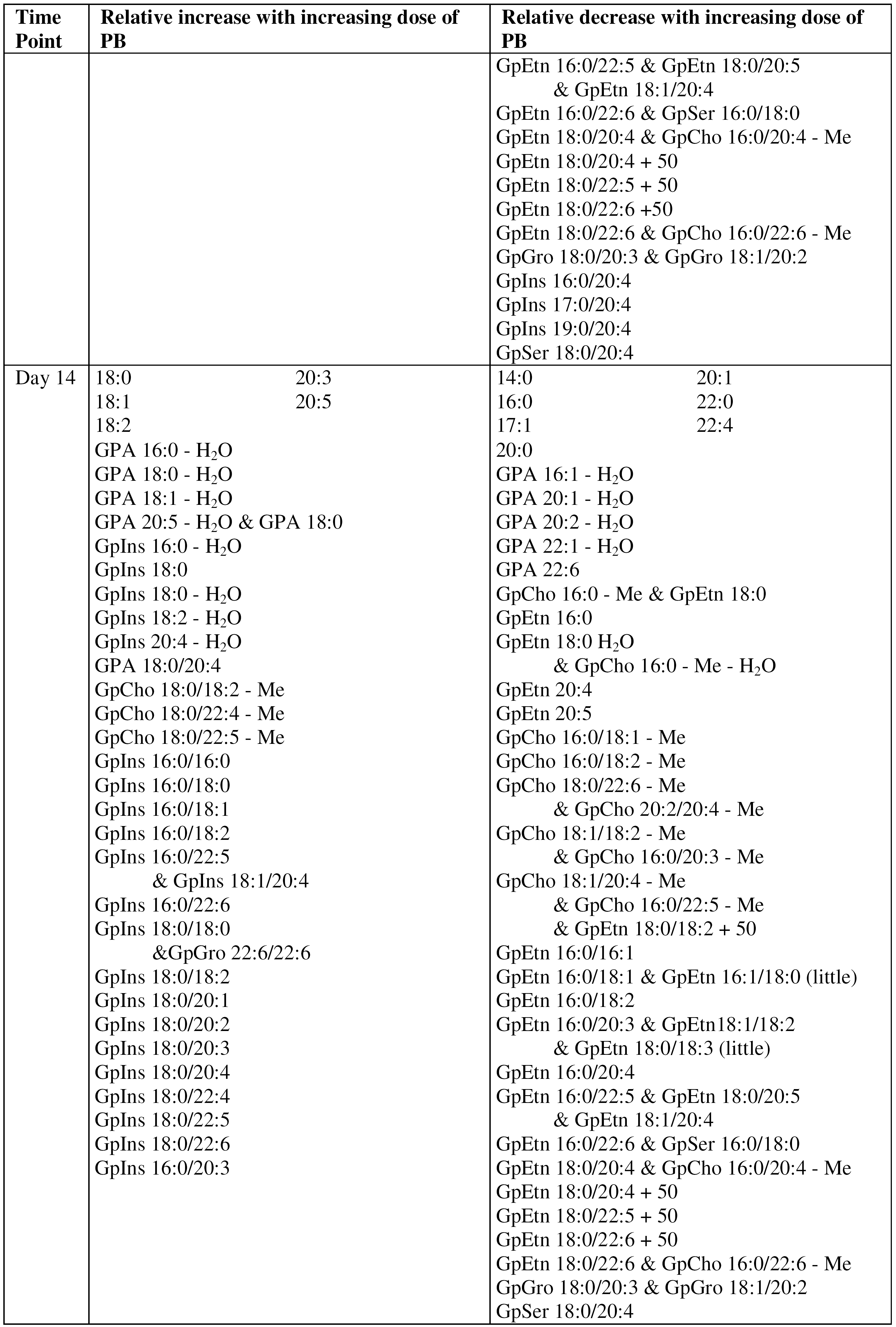

Supplement: Additional file 3 — Remodelling of the lipid profile of liver tissue following PB treatment. Changes in intact phospholipids in response to increasing dose of PB as detected by negative mode direct infusion ESI-MS of extracts from liver tissue. [file 1471-2164-11-9-S3.DOC]
